# 造血干细胞移植50年——从拓荒到领航，中国方案重塑全球格局

**DOI:** 10.3760/cma.j.cn121090-20250509-00221

**Published:** 2025-06

**Authors:** 晓军 黄

**Affiliations:** 北京大学人民医院，北京大学血液病研究所，国家血液系统疾病临床医学研究中心，造血干细胞移植治疗血液病北京市重点实验室，北京 100044 Peking University People's Hospital & Peking University Institute of Hematology, National Clinical Research Center for Hematologic Disease, Beijing Key Laboratory of Hematopoietic Stem Cell Transplantation, Beijing 100044, China

## Abstract

造血干细胞移植作为治愈大多数血液系统疾病的有效手段，于中国的发展历程堪称辉煌。1964年，陆道培成功完成亚洲首例、全球第四例造血干细胞移植术，开启中国在该领域的征程。2000年，黄晓军完成世界首例非体外去T细胞单倍型相合造血干细胞移植术，并持续优化，使其体系在2016年被世界骨髓移植协会命名为“北京方案”，如今成为全球单倍型移植的主流方案。新型细胞治疗的出现革新了传统造血干细胞移植模式。中国学者从学习西方起步，一路奋进，重塑了全球造血干细胞移植格局。

## Abstract (en)

Hematopoietic stem cell transplantation（HSCT）, a curative procedure for the majority of hematologic disorders, has seen remarkable development in China. In 1964, Lu Daopei performed Asia's first and the world's fourth HSCT, marking the beginning of China's progress in this field. Huang Xiaojun completed the world's first haploidentical HSCT without in vitro T-cell depletion in 2000. The protocol has evolved into a system named “Beijing Protocol” by the Worldwide Network for Blood and Marrow Transplantation（WBMT）in 2016, which is currently a mainstream global haploidentical transplantation protocol. The emergence of novel cellular therapies has revolutionized the conventional hematopoietic stem cell transplantation model. From learning from the west, Chinese scholars have strived to reshape the global HSCT landscape.

造血干细胞移植是大多数血液系统疾病的有效治愈方式，在过去50年间，中国在造血干细胞移植领域取得了举世瞩目的成就。1957年，Thomas首次报道了现代造血干细胞移植模式。1964年，陆道培完成了亚洲第一例、全球第四例造血干细胞移植术，开创了中国造血干细胞移植的先河。2000年，黄晓军完成世界第一例非体外去T细胞单倍型相合造血干细胞移植术，随后不断优化丰富该单倍型移植体系，2016年世界骨髓移植协会将该体系命名为“北京方案”，“北京方案”现已成为全球治疗单倍型相合造血干细胞移植的主流方案。从最初的学习西方先进科学技术，到如今为世界同行制定行业标准，中国学者不仅实现了自身从无到有、从弱到强的跨越，更是重塑了全球造血干细胞移植格局。

一、破冰移植技术，西方探索引领全球发展

1939年，人类历史上首例骨髓移植应用于重金属引发的再生障碍性贫血患者。临床医师凭借有限的认知和勇于探索的精神，为患者静脉输注了ABO血型相合兄弟的骨髓，虽以失败告终，却开启了移植医学的探索之路。1957年，美国科学家Thomas首次探索现代造血干细胞移植模式，最初的尝试中，6例接受移植的患者仅有1例存活超过100 d，其余均在短期内死亡[1]。直到1977年，100例化疗失败的急性白血病患者接受HLA相合同胞异基因造血干细胞移植（allo-HSCT），13例患者获得长期生存，由此揭开造血干细胞移植治愈恶性血液病的绚烂序章。与此同时，自体造血干细胞移植也在1977年取得了重大突破，首次成功用于治疗复发性急性髓系白血病（AML）[2]，并逐步扩展至非霍奇金淋巴瘤和多发性骨髓瘤。随着对HLA理解的深入、抗原鉴定技术的提高以及对造血干细胞移植需求的增加，1980年Thomas进行了HLA配型相同的非血缘供者骨髓移植，证明了非亲属间骨髓移植的可行性[3]。1990年，Thomas由于在人体器官和细胞移植研究中的贡献而获得诺贝尔生理学或医学奖。

西方学者率先开启造血干细胞移植的探索之门，中国学者亦迅速跟进，在这一前沿阵地崭露头角。1964年，陆道培在北京大学人民医院成功完成亚洲第一例、世界第四例异体同基因骨髓造血干细胞移植，这一里程碑式的成果标志着我国造血干细胞移植事业的诞生。1981年，陆道培成功进行了我国首例同胞相合allo-HSCT，为我国造血干细胞移植技术的发展积累了宝贵经验。1986年，严文伟带领团队成功实施了国内第一例自体骨髓造血干细胞移植，开辟了我国自体骨髓造血干细胞移植治疗恶性血液病的新领域。

二、攻克供者瓶颈，北京方案重塑世界格局

在2000年以前，全球造血干细胞移植技术的发展受制于HLA相合供者匮乏。中国约90%患者需移植但无HLA配型相合的供者，欧美国家40%～50%患者因供者来源问题无法进行移植，因此供者来源匮乏是当时限制移植应用的世界性难题。单倍型相合移植历经艰辛探索，尽管体外去除T细胞（TCD）和大剂量CD34^+^造血干细胞的“意大利方案”减少移植物抗宿主病（GVHD），但该方案治疗后的复发、感染风险仍居高不下[4]。

爱默生曾说过“困难是勇敢者前进的脚踏石”。面对我国供者来源尤为突出的境况，自20世纪90年代起，黄晓军致力于探索粒细胞集落刺激因子（G-CSF）诱导T细胞免疫耐受研究，首创G-CSF动员的供者干细胞采集物联合受者应用抗胸腺细胞球蛋白（ATG）的临床免疫耐受新方案，2000年成功完成第1例同胞半相合造血干细胞移植；2002年，第1例父母半相合造血干细胞移植取得成功；系列临床研究证实，此方案可将半相合移植急性GVHD发生率降至20%～40%[5]–[7]。随后黄晓军等围绕半相合移植开展了系列创新研究，包括稳定植入、供者优选、复发及感染防治等。2016年世界骨髓移植协会主席将这一系统性工作称为“北京方案”[8]。“北京方案”使半相合移植治疗白血病的3年生存率从约20%升至70%左右，在全球首次实现半相合移植疗效优于化疗，首次实现半相合移植与全相合移植疗效相同[9]–[11]；改变了世界半相合移植“不可逾越”的传统观念，使半相合移植成为白血病的一线治疗方案[12]。欧洲血液学会主席Fibbe等评价称“‘北京方案’为缺乏全相合供者的可靠替代方案”，“已成为全球半相合移植新标准”。“北京方案”解决了供者来源匮乏的世界性难题，使半相合移植成为白血病的一线治疗方案，成为全球半相合移植新标准，引领全球进入“人人都有造血干细胞移植供者”的历史新时代。

2008年，美国约翰霍普金斯大学率先报道了另一种GVHD预防方案，基于移植后大剂量环磷酰胺（PTCy）使单倍型相合移植GVHD发生率可接受、生存率有所提高[13]。近年来，该PTCy方案与小剂量ATG的联合治疗策略进一步改善了移植疗效。基于中国造血干细胞移植登记组（CBMTRG）数据的配对研究比较了G-CSF/ATG方案、PTCy方案及PTCy联合低剂量ATG方案在血液系统恶性肿瘤患者中的临床疗效，发现接受G-CSF/ATG方案治疗患者造血重建更快、生存期更长，更具临床优势[14]。目前，非体外去T细胞的“北京方案”和“美国方案”已成为国际主流单倍型相合移植方案。

“北京方案”关键技术的广泛应用推动全球造血干细胞移植的快速发展。半相合移植现已成为我国造血干细胞移植的主要模式，年移植病例数从2008年的337例增长到2023年的9 908例，总移植病例数已超过5.5万例，其中94%采用了“北京方案”新模式。全国移植中心数量由2008年的27个增长至2023年的212个[15]；得益于“北京方案”的成功实施，北京大学人民医院已成为全球最大的造血干细胞移植中心，2023年完成半相合移植超过1 000例。“北京方案”的关键技术还推广至韩国、意大利、法国等十余个国家和地区，使全球超过半数的单倍型相合移植应用“北京方案”[16]。“北京方案”的成果不仅改写了43项国际移植指南和共识，还被纳入以诺贝尔奖获得者命名的国际权威教科书《托马斯造血干细胞移植》的第四版和第五版，成为“中国技术改变世界格局的典范”（引自第二届转化医学奖评述）。

三、融合细胞免疫，协同治疗革新移植模式

创新治疗方法大量涌现，嵌合抗原受体T细胞（CAR-T细胞）疗法等治疗均对造血干细胞移植时机及移植模式产生了重要影响。2011年美国率先开启CAR-T细胞免疫治疗的临床应用，CAR-T细胞疗法的先驱June、Rosenberg、Sadelain首次使用CAR-T细胞免疫疗法成功治疗一例复发急性B淋巴细胞白血病儿童。随后中国学者紧跟时代步伐，在CAR-T细胞免疫治疗领域同样取得了令人瞩目的进展，从靶点应用到细胞构建，再到临床应用的创新，均取得了显著成果。2014年，韩卫东团队报道了利用CD20 CAR-T细胞技术治疗难治弥漫大B细胞淋巴瘤的初步结果，这是中国在CAR-T细胞免疫疗法领域的首次探索报道[17]。

近年来，我国CAR-T细胞治疗血液系统恶性疾病的临床研究表现活跃，其发展创新聚焦于新型靶点、功能强化、疾病拓展、融合移植新模式等。如新型T细胞受体-抗原受体（STAR）整合OX40共刺激结构域（即STAR-OX40）后显著增强了T细胞的增殖和持久性。2022年，陆佩华团队首次报道了STAR-OX40 T细胞治疗18例复发难治急性B淋巴细胞白血病（B-ALL）的临床研究，所有患者输注后4周内实现完全缓解，表明这是一种安全性较高且有前景的治疗方法[18]。在CD38 CAR-T细胞治疗复发难治AML方面，吴德沛、唐晓文团队探索了6例allo-HSCT后复发AML患者接受靶向CD38 CAR-T细胞治疗后的疗效和安全性，缓解率达66.7%以上，且未引发严重的细胞因子释放综合征（CRS）和免疫效应细胞相关神经毒性综合征（ICANS）[19]。2022年，陆佩华与黄晓军共同报道了一种无需额外基因编辑的“自然杀伤”CD7 CAR-T细胞疗法（NS7CAR）在人体试验中的应用，结果表明，20例患者中19例骨髓达到微小残留病阴性完全缓解，证实NS7CAR对于治疗难治复发急性T淋巴细胞白血病/T淋巴母细胞淋巴瘤安全且高效[20]。同年，黄河团队通过基因编辑技术解决了免疫排斥等难题，成功构建了健康供者来源的靶向CD7通用型CAR-T细胞（RD13-01），并完成了全球首个RD13-01Ⅰ期临床研究[21]。2024年，黄河团队提出了CAR-T细胞治疗与allo-HSCT一体化的序贯治疗方案，这种创新性一体化策略无需传统清髓预处理和GVHD预防药物，为难治复发CD7阳性血液系统疾病患者提供了全新选择[22]。上述创新性研究为传统治愈手段与前沿免疫细胞治疗的联合应用提供了新思路，不断夯实了“移植+免疫”联合治疗的临床价值。

因此，新型细胞治疗如CAR-T细胞治疗既为更多难治复发疾病患者创造了造血干细胞移植桥接时机，并革新了移植模式。我国学者在CAR-T细胞免疫治疗领域的持续探索和创新实现了从“跟跑”到“并跑”的跨越，不仅为血液病患者带来了更多治疗选择，也为全球CAR-T细胞治疗的发展贡献了中国智慧。但目前中国批准上市的CAR-T细胞产品主要通过技术引进和合作生产模式，尚未形成完全自主的原创制备技术。

四、探索精准靶向，多元发展开辟全新纪元

在医学发展迭代升级的浪潮中，精准治疗已成为未来重要航行方向。精准造血干细胞移植的内涵包括预处理方案个体化、移植合并症预测化等，个性化精准治疗有望为更多血液病患者带来更显著的疗效。

在精准造血干细胞移植领域，优化预处理方案是提高移植成功率和患者生存率的关键策略之一。针对高龄及并发症患者的特殊需求，通过探索创新药物组合与剂量调控策略，为移植安全性与有效性筑牢根基。北京大学人民医院牵头的一项前瞻性研究显示，高龄（≥55岁）急性白血病或骨髓增生异常综合征患者单倍型移植采用了减低剂量环磷酰胺联合氟达拉滨、ATG和白消安的新型预处理方案，移植后1年无白血病生存率为60.2%，倾向评分匹配方法证实该结果与接受Bu/Cy/ATG方案的单倍型移植患者相似[23]，证明了个性化预处理方案在高龄单倍型移植患者中的可行性和有效性。在个性化预处理方案的保驾下，北京大学人民医院创造了allo-HSCT受者最高年龄77岁的亚洲纪录。

以G-CSF/ATG为基础的非体外去T细胞方案使单倍型移植成功跨越HLA屏障，从治疗白血病拓展至再生障碍性贫血、范可尼贫血、阵发性睡眠性血红蛋白尿、遗传代谢性疾病等非恶性疾病。不同疾病的预处理方案不同，合并不同危险因素的同一疾病预处理方案亦不同。以再生障碍性贫血为例，黄晓军团队在全球首次通过前瞻性研究报道单倍型相合移植应用白消安、环磷酰胺200 mg/kg和ATG预处理方案治疗重型再生障碍性贫血：存活者移植后生活质量评分逐年增高，9年生存率达85%[24]；登记组数据比较证明，该方案可获得与同胞相合移植一致的疗效[25]；新英格兰杂志评述称之为“令人鼓舞的中国经验”[26]。尽管生存率高，该预处理方案仍有心脏不良事件发生，北京大学人民医院创建心脏不良事件预测模型，优化高危人群预处理方案，使移植后早期严重心脏不良反应发生率从12.8%降至2.2%，提高了移植的安全性[27]，并建立高龄再生障碍性贫血allo-HSCT生存的预测模型，进一步明确获益于allo-HSCT的高龄再生障碍性贫血患者特点[28]–[29]。

如今，在人工智能大背景下，创建移植合并症预测模型并进行精准干预是发展方向。以移植后复发为例，北京大学人民医院团队创建了AML单倍型移植后复发的计算模型，复发高危人群可进行干扰素等免疫治疗预防复发[30]。此外，黄晓军团队率先建立了G-CSF动员的供者淋巴细胞联合短程免疫抑制剂新方案治疗移植后微小残留病阳性患者，3年复发率由50%～64%降至21%～28%[31]；创建了强化预处理联合微小残留病和GVHD双标记指导复发干预的整体治疗新策略，使难治/复发白血病3年无病生存率由0～35%升至50%～56%[32]。近年来，靶向BCR∷ABL、FLT3、IDH1/2、BCL2等小分子药物、靶向CD19/CD3双特异性抗体如Blinatumomab等免疫治疗抗体药物不断涌现，可应用于移植前桥接、移植预处理及移植后维持等，由此助力造血干细胞移植实现血液肿瘤患者无病长生存。

从20世纪到21世纪，造血干细胞移植迎来了从创立、发展到完善的巨大飞跃。而过去50年是中国造血干细胞移植技术蓬勃发展的黄金时代，其发展进程宛如一部镌刻着挑战与突破的医学史诗。从最初的艰难探索到如今的创新发展，“北京方案”等中国原创成果已在全球医学舞台上绽放璀璨光彩，成为中国医学智慧的标志性符号。在不断前行的浪潮中，靶向治疗与免疫治疗的深度融合成为新引擎，为造血干细胞移植注入澎湃动力。未来人工智能与大数据的深度赋能，将推动造血干细胞移植治疗迈向前所未有的新高度，为血液肿瘤患者带来更光明的未来。
